# Monogenic and polygenic concepts in chronic kidney disease (CKD)

**DOI:** 10.1007/s40620-023-01804-8

**Published:** 2023-11-21

**Authors:** Julia Jefferis, Rebecca Hudson, Paul Lacaze, Andrew Bakshi, Carmel Hawley, Chirag Patel, Andrew Mallett

**Affiliations:** 1 Genetic Health Queensland, Royal Brisbane and Women’s Hospital, Brisbane, QLD Australia; 2 Faculty of Medicine, University of Queensland, Brisbane, Australia; 3 Kidney Health Service, Royal Brisbane and Women’s Hospital, Brisbane, Australia; 4 School of Public Health and Preventive Medicine, Monash University, Melbourne, VIC Australia; 5 Department of Nephrology, Princess Alexandra Hospital, Woolloongabba, QLD Australia; 6 Australasian Kidney Trials Network, The University of Queensland, Brisbane, QLD Australia; 7 Translational Research Institute, Brisbane, QLD Australia; 8 Institutional for Molecular Bioscience and Faculty of Medicine, The University of Queensland, Saint Lucia, Australia; 9 Department of Renal Medicine, Townsville University Hospital, Douglas, QLD Australia; 10 College of Medicine and Dentistry, James Cook University, Douglas, QLD Australia

**Keywords:** Kidney function, Heritability, Polygenic risk score, Membranous nephropathy, IgA nephropathy, Hypertension

## Abstract

Kidney function is strongly influenced by genetic factors with both monogenic and polygenic factors contributing to kidney function. Monogenic disorders with primarily autosomal dominant inheritance patterns account for 10% of adult and 50% of paediatric kidney diseases. However, kidney function is also a complex trait with polygenic architecture, where genetic factors interact with environment and lifestyle factors. Family studies suggest that kidney function has significant heritability at 35–69%, capturing complexities of the genome with shared environmental factors. Genome-wide association studies estimate the single nucleotide polymorphism-based heritability of kidney function between 7.1 and 20.3%. These heritability estimates, measuring the extent to which genetic variation contributes to CKD risk, indicate a strong genetic contribution. Polygenic Risk Scores have recently been developed for chronic kidney disease and kidney function, and validated in large populations. Polygenic Risk Scores show correlation with kidney function but lack the specificity to predict individual-level changes in kidney function. Certain kidney diseases, such as membranous nephropathy and IgA nephropathy that have significant genetic components, may benefit most from polygenic risk scores for improved risk stratification. Genetic studies of kidney function also provide a potential avenue for the development of more targeted therapies and interventions. Understanding the development and validation of genomic scores is required to guide their implementation and identify the most appropriate potential implications in clinical practice. In this review, we provide an overview of the heritability of kidney function traits in population studies, explore both monogenic and polygenic concepts in kidney disease, with a focus on recently developed polygenic risk scores in kidney function and chronic kidney disease, and review specific diseases which are most amenable to incorporation of genomic scores.

**Graphical abstract:**

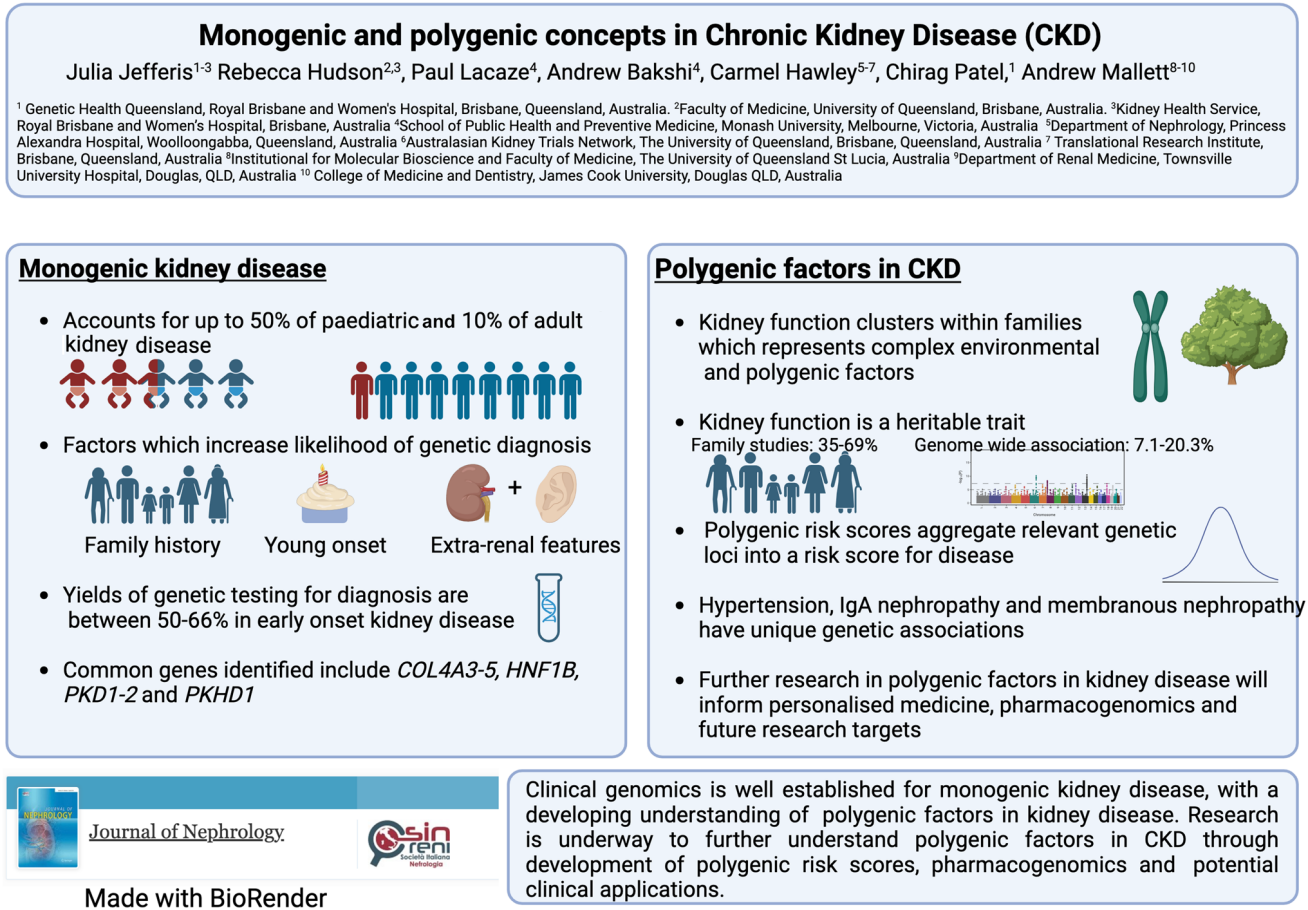

## Take-home messages

**Table:** 

Kidney function has significant genetic determinants from both monogenic and polygenic factors.
Family studies estimate heritability of kidney function at 35–69% which captures complex genetic architecture but underestimates shared environmental factors in families.
Genome-wide association studies estimate heritability of kidney function between 7.1 and 20.3%, but are higher in targeted disease states such as diabetes and differ across ethnicities.
Polygenic risk scores estimate genetic risk from GWAS and correlate well with kidney function at a population level but not at an individual level, with environmental factors having an important role.
Polygenic risk scores ascertain the genetic risk for IgA nephropathy and membranous nephropathy and provide extra information that could be integrated into established risk scores to improve diagnosis and prognostication.
Polygenic risk scores at a population level have identified common genes which may help elucidate common disease pathways in the development and progression of kidney disease.
Incorporating genomics in clinical practice is underway with focus on personalised medicine, pharmacogenomics and identifying common pathways in kidney disease as treatment targets.

## Introduction

Genomics is a rapidly expanding field in medicine. Kidney disease has a strong genetic basis and we are beginning to understand the complexities of both its monogenic (single-gene) and polygenic (multi-gene) forms. Recent population studies have found that kidney function has a significant polygenic component, separate from the more traditionally known forms of monogenic kidney disease [1, 2]. A polygenic view of kidney function has raised new questions in nephrology, warranting further investigation to determine the clinical relevance and application of polygenic risk scores.

Kidney function is highly heritable, and is considered a complex trait, where both genetic and environmental factors contribute [3]. Kidney function, most commonly assessed by estimated glomerular filtration rate (eGFR), chronic kidney disease (CKD) stage, albuminuria or proteinuria, is impacted by an array of disease states, all of which determine kidney health in individuals across their life spans [4]. Heritability estimates the amount of variation in a trait which is determined by genetic factors, and can be considered an indicator of genetic predisposition to developing a disease [5]. A key question is to what extent kidney function is determined by genetic differences, and how this knowledge might impact clinical practice.

Certain kidney diseases display Mendelian and monogenic inheritance patterns. These rarer diseases are most commonly observed in younger patients, including Autosomal Dominant Polycystic Kidney Disease (ADPKD), Alport syndrome, autosomal recessive disorders such as nephronophthisis, and inherited forms of atypical haemolytic uraemic syndrome which can have both monogenic inheritance (*DGKE, CFH)* or genetic predisposition (*CFHR3-5del*) [6–9]. These rare diseases explain approximately 50% of paediatric and 10% of adult CKD, yet we clearly see clusters of kidney disease amongst families and ethnicities, highlighting potential genetic predisposition and environmental determinants of disease [6, 10, 11].

**Fig. 1:**
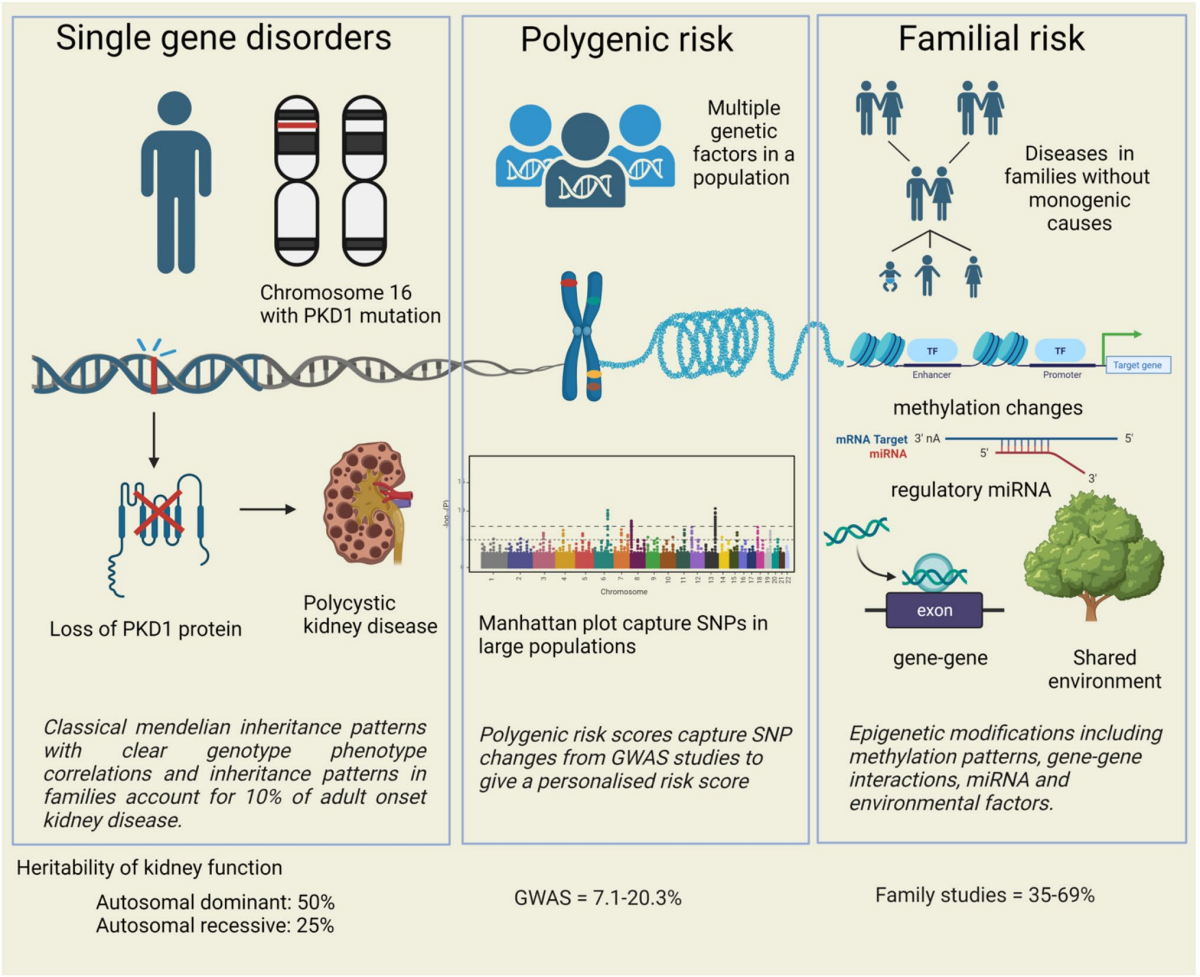
Concepts of heritability in monogenic, polygenic and familial patterns of kidney disease. Created with BioRender.com

In parallel, Genome-Wide Association studies (GWAS) have reported the single nucleotide polymorphism- (SNP) based heritability of CKD to be between 7.1 and 20.3%. This estimate is lower than that observed in familial studies of CKD, where heritability estimates have ranged between 35 and 69% (Fig. 1) [2, 12–14]. Genome-wide association studies only capture additive variances in genetic sequences, whereas population studies encapsulate more complex genomic structures including epigenetic factors which yield higher heritability estimates. Familial heritability estimates diminish with age, suggesting that at older ages, modifiable risk factors are more important [15]. Polygenic risk scores are derived from GWAS and use disease-associated SNPs across the genome to estimate risk. Polygenic risk scores aggregate signals from many different genetic loci into a single score and measure genetic risk based on common variants. Recently, polygenic risk scores for kidney function have been derived and validated in large studies. [1, 16–19] However, these polygenic risk scores do not measure or account for rare genetic variation, and still require further development and assessment to guide clinical translation. Common kidney diseases such as IgA and membranous nephropathy have significant genetic risk, and polygenic risk scores may be useful in these specific diseases [20, 21]. Hypertension is a strong risk factor for CKD, and has its own unique polygenic risk score with hundreds of possible loci, which only have a small impact on blood pressure and are separate from CKD risk [22]. This review will explore genetic factors in kidney disease, considering family studies, monogenic disorders, GWAS, and polygenic risk scores, with exploration of genetic factors in specific disease states. It will give an oversight of the genetic interplay with kidney disease, and relevant clinical applications. Understanding the complex genetic architecture of kidney disease will be important for the development of future diagnostic, therapeutic and preventive strategies.

## Kidney disease in families

Family history of kidney disease is often the first clue to both monogenic and polygenic components in CKD. Family history can preclude the perceived need for genetic testing in diseases such as ADPKD and Alport syndrome, where the clinical phenotype coupled with a family history, cement the diagnosis and enable appropriate counselling and screening for family members [23, 24]. Interestingly, large cross sectional studies from Norway have shown that traditionally non-genetic kidney diseases cluster in families. Examples include glomerular disease, interstitial nephritis and hypertensive nephrosclerosis, with a relative risk (RR) of 3.7 (95% CI 3.1, 4.4) in first degree family members of those with kidney failure [11]. The Lifelines cohort study spanned three generations, and determined the RR of 3.0 (95% CI 2.3, 4.1) for kidney disease in those with an affected first degree relative, which suggests strong genetic factors for CKD. They also found a RR for kidney disease in those with an affected spouse of 1.6 (95% CI 1.20, 1.96), suggesting shared environmental factors also impact CKD, highlighting that families may have both shared genetic and environmental factors, such that it can be hard to distinguish pure genetic contributions [25]. Potential application of family history includes screening family members of dialysis patients to identify those at heightened risk of CKD and kidney failure, however there have been challenges to implementing this in clinical practice. One study identified 26.2% of those screened with a new diagnosis of proteinuria, though a key limitation of the study was that recruitment was through dialysis populations, thereby limiting the study to individuals able to access screening measures within the health care system [26]. In Saudi Arabia, screening family members of dialysis patients identified 5.8% of family members with an eGFR < 60 ml/min/1.73 m^2^ and 8% with new proteinuria, suggesting the usefulness of targeting family members of those with kidney failure as a targeted screening program to identify early CKD [27]. Family history is a key component of work-up of CKD patients with implications for both the patient and their family, with the caveat that it is subject to recall bias and ability to obtain an accurate family history and clinical details.

### Monogenic disorders and diagnostic testing

High numbers of monogenic kidney diseases are identified through genetic testing with clinically meaningful impacts for patients. Monogenic disorders are commonly diagnosed in both children and adults with kidney disease. Gene panel testing is available and can have diagnostic utility in 50–66% of early onset kidney disease, depending on the population studied [28]. A study in patients with unexplained kidney disease with onset before 30 years of age found 65% had genetic kidney disease, and that 66% of these genetic diagnoses were explained by seven key genes (*COL4A3, COL4A4, COL4A5, HNF1B, PKD1, PKD2* and *PKHD1*), with only 49% having family history of kidney disease [29]. In Ireland, targeting patients with family history of CKD, ‘extra-renal features’ or uncertain aetiology was able to identify a relevant genetic result in 50.9% of patients, with a higher yield of 67.2% in those with a positive family history. The most common causes identified were related to the *PKD1*, *PKD2*, *MUC1* and *COL4A5* genes, with a further 36 genes reported [30]. A recently published systematic review of genetic testing in kidney disease cohorts identified that risk factors for genetic kidney disease include positive family history, consanguinity, extra-kidney features, early onset disease and kidney failure at any age, and highlighted the importance of testing for both copy number variants and single nucleotide variants to improve diagnostic yield. Importantly, genetic testing enables personalised medicine in areas of diagnosis, treatment and family impacts [31]. Genetic testing in patients with unexplained kidney disease can have a significant diagnostic yield, between 17 and 47%, suggesting genetic causes of CKD should be considered in these cohorts. Genetic diagnosis in monogenic CKD has numerous clinical implications, and studies show that developing a clinical work flow can facilitate a genetic diagnosis in two thirds of high risk patients, with modelling showing a reduction in diagnostic costs by 20% when integrated early [32]. Improving clinician awareness of genetic kidney disorders, particularly the variable clinical manifestations of genetic kidney disease, and education to improve understanding of genetic testing will enable integration into mainstream clinical practice to directly improve patient outcomes [33]. These include ensuring correct diagnosis, changes to prognosis, avoidance of unnecessary therapies or intervention, need for screening for associated disease states, assisting family planning and guiding living related donors in transplantation.

### Environment and genetics

Established environmental factors contributing to CKD include socioeconomic status, occupational exposure, cigarette smoking and infections [3, 34]. Internationally, geographical regions associated with higher rates of CKD are purported to represent environmental factors, however the clustering in certain populations also suggests genetic susceptibility to disease [35]. Sri Lanka has high rates of tubulointerstitial CKD in agricultural populations, which clusters in families, and is not explained by traditional factors such as diabetes or hypertension. Genome-wide association studies identified *SLC13A3*, a gene which encodes a sodium dicarboxylate transporter in the proximal tubule, carrying a 50% increased risk for CKD through a yet unknown mechanism, and is postulated to interact with a yet unknown environmental trigger [36].

*APOL1* high-risk alleles have been implicated in focal segmental glomerulosclerosis, HIV-associated nephropathy, lupus nephritis and CKD. The *APOL1* gene has two risk alleles, G1 and G2, that are prevalent in persons of sub-Saharan African descent, and to which protection against trypanosomes has been attributed. *APOL1* risk alleles are associated with increased risk of kidney diseases, but not all people with these alleles do develop disease, suggesting possible complex genomic and environmental triggers, particularly for development of hypertension or HIV nephropathy.[37–39]. Carrying two risk alleles for *APOL1* is associated with an 89% increased risk of HIV-associated nephropathy compared to HIV-positive controls without these risk alleles [40]. There is controversy in the clinical use of *APOL1*, as it can be difficult to determine the significance of ancestry and alleles from socioeconomic status and race in African-American populations [41]. APOL1 status may have clinical implications in management and kidney donation, which needs to be approached sensitively [41]. A randomized control trial in 2050 patients of African ancestry with hypertension found that early disclosure of *APOL1* genotype led to improvement in systolic blood pressure control in high risk groups, presumably due to better patient engagement, and improved screening for proteinuria, however long-term data are still lacking [42]. Consensus opinion promotes testing *APOL1* in potential living kidney donors of African descent to guide donor risk [43]. The complexity of determining genetic contributions to a complex disease state such as CKD are challenging, however utility and clinical implications for patients is paramount, as genetic diagnosis can aid both patients and families.

### Familial studies

Familial studies across several generations show clustering of kidney function without a monogenic cause suggesting multiple polygenic influences [25]. Twin studies are a natural model to assess genetic and environmental determinants of health, through studying monozygotic (100% shared genetic material) and dizygotic (50% shared genetic material) twins with exposure to the same environmental factors. Twin studies have estimated heritability of kidney function ranging from 18 to 76%, which diminishes with age, suggesting environmental determinants are more important in older age, with an underlying genetic component [15]. Family studies which include sibling and parent relationships have a similar variability in heritability, estimating heritability of kidney function between 35 and 69%. Risk factors for CKD, such as hypertension and diabetes, also cluster in families, and can increase CKD risk. Heritability estimates of kidney function in hypertensive families of African descent is 51%, again highlighting genetic contributions although confounded by shared environmental factors amongst families [10]. Diabetic kidney disease congregates in families, suggesting both genetic and environmental factors drive disease [44, 45]. A study of 662 diabetic participants from 310 families estimated heritability of eGFR at 75% and albuminuria at 46%; clearly this is in this high risk population [14]. Family studies enable assessment of targeted populations and theoretically capture all familial genetic and epigenetic factors, but tend to over-estimate genetic contributions to a trait and underestimate environmental determinants.

### GWAS

Genome-wide association studies are large scale studies able to determine genetic variants associated with a phenotype including eGFR, CKD status and albuminuria. A UK BioBank study assessed 35 traits in 363,228 individuals and estimated heritability, the proportion of the phenotype explained by genetic factors, at 20.3% for eGFR but only 3.3% for albuminuria [2]. Interestingly, a targeted GWAS in diabetic patients identified a locus in *GABRR1* associated with albuminuria in diabetic patients, which was only identified in European populations and not replicated in Asian populations [46]. This highlights that GWAS findings critically depend on the population studied, with ancestry impacting genetic loci identified. Genome-wide association studies for CKD have been performed predominantly in European ancestry populations, but the expansion to other populations has led to the discovery of other genetic determinants [12]. Genome-wide association studies enable identification of genetic variants related to kidney function, and are targets for further investigation into associations with clinical disease or potential therapeutic targets. One of the largest GWAS, involving 1,046,070 individuals, identified 264 key SNPs associated with eGFR, with heritability estimated at 19.6% [12]. Co-localisation studies found differential tissue expression of 17 genes, in both causal and regulatory pathways, which require further research into common pathways contributing to kidney disease. An example is SNP rs11919484, identified in this GWAS which localised to *KNG1* and co-localisation studies found expression in the kidney tubulointerstitium, with a biological mechanism associated with the renin-angiotensin system, thus a plausible disease susceptibility gene which warrants further study in CKD [12]. Additionally, targeting small populations highly affected with disease will identify different genetic factors than those found in healthy populations, such as identification of *KCNIP4* locus in the CKD population in Norfolk Island [47]. Genome-wide association studies enable identification of novel genes and drug targets and are relatively cost effective, however limitations include identification of genes with no biological function, inability to capture gene–gene interactions, limited representation of non-European ancestries, and failure to account for environmental factors. Results should be interpreted appreciating these important aspects [48]. An important limitation in these large GWAS studies is that kidney function is determined by the widely available, but more variable, estimated GFR than the more precise measured GFR. Furthermore, there are limited studies assessing polygenic risk scores for highly relevant kidney function traits including albuminuria, rate of decline in eGFR and development of kidney failure (Table 1).

**Table 1:** Summary of key GWAS for kidney associated phenotypes

First author	PMID	Year	Population	Number	Ancestry	Trait	Heritability	Comments
Wuttke	31152163	2019	Multi-ancestry registry CKD Gen consortium	1,046,070	European (54%), East Asian (15.8%), African-American (1.3%), South Asian (1.28%), Hispanic (0.47%)	eGFR	0.20	Multi-ancestry population, mean eGFR 89 ml/min/1.73 m^2^, inability to capture low frequency or population specific variants
Sinnott-Armstrong	33462484	2021	UK Biobank registry	363,228	European (100%)	eGFR Cystatin C Urea Microalbuminuria	0.20 0.21 0.12 0.03	Assesses kidney function in a large European population. Translation to other ancestries is, attempts to reduce bias may reduce power of detection of rare SNPs
Liu	35710981	2022	CKDGen, Pan-UK Biobank, MPV, PAGE, Summit consortia	1,500,000	European (80%), East Asian (11%) African (4%) Hispanic/Latino (1.6%) African-American (1.5%) Central/South Asian (1.4%) Middle Eastern (0.10%) Admixed American (0.06%) Native American (0.04%) Asian American (0.01%) Other (0.03%)	eGFR (creatinine) eGFR (methylation)	0.07 0.21	Largest analysis across multiple ancestries incorporating methylation data
Van Zuydem	29703844	2018	Multi-registry study with diabetic kidney disease	5717	Asian and European	eGFR CKD Diabetic kidney disease	0.07 0.12 0.08	Spectrum of diabetes and diabetic kidney disease from multi-national studies, limited by small numbers. Lower heritability estimates in population enriched for disease
Kirkyluk	25305756	2014	Biopsy-confirmed IgA nephropathy	6699	European (68%) Han Chinese (32%)	IgA nephropathy	NA	Identification of 6 loci associated with IgA
Xie	32231244	2020	9 case control cohorts for Membranous Nephropathy	3782 cases and 9038 controls	European (62%) East Asian (38%)	IgA	0.36 0.43	Discovery cohort enriched with membranous nephropathy, applicability to broader population limited
Ehret	21909115	2011	Hypertension	200,000	European	Systolic and diastolic BP	NA	Identified 116 variants associated with hypertension, explains 2.2% variance in blood pressure

### Heritability gap

The heritability gap between family (observational) and GWAS (inferential) studies is exemplified in CKD, where the estimation of the proportion of kidney function explained by genetic factors is much higher in twin and familial studies (18–76%) than in GWAS (7.1–20.3%). Family studies underestimate the contribution of shared environmental factors leading to higher heritability estimates, but are also subject to ascertainment bias in families. Genome-wide association studies have lower heritability estimates as they rely on SNPs that meet thresholds of *p* < 5 × 10^8^, but this can be improved by incorporating machine learning and phenotype correlation at lower significance thresholds to improve identification of less common alleles associated with rare diseases [5]. Genome-wide association study data reduce the complex structure of DNA to single nucleotide polymorphisms, and do not account for the complexities of genetic architecture, nor epigenetic modifications, which are captured in family studies [49]. In a large scale twin study, heritability estimates for eGFR from SNP data were 32%, whereas traditional twin estimates of heritability were marginally higher at 38%, again showing higher heritability estimates from family studies for kidney function [50]. Genome-wide association studies which have incorporated methylation data into their analysis of heritability, propose that methylation variants explain a larger proportion of variance in creatinine-based eGFR than SNP data, with genome changes explaining 21% of heritability but methylation changes accounting for 41% of phenotypic variation [51]. Other epigenetic factors that may explain this heritability gap include differential expression of miRNA in kidney disease. A case control study in 15 pairs from the Atherosclerosis Risk in Communities (ARIC) cohort found downregulation of miR-15 and miR-17 in individuals with CKD and treated hypertension, although interestingly these are associated with immune cell activity [52]. This suggests that while GWAS are useful, our understanding of complex genetic architecture is lacking, and this is where family studies are still relevant to help understand heritability of traits, as shown in Fig. 1.

## Polygenic Risk Scores

Polygenic risk scores can be derived from GWAS for a variety of clinical phenotypes, and are typically calculated by summing the estimated genetic risk from a set of multiple independent SNPs into a single risk score. Several polygenic risk scores have been developed for kidney traits such as eGFR, CKD stage, AKI and kidney failure [18]. A multi-ancestry polygenic risk score was developed across several populations and found that CKD status was associated with polygenic risk scores in European (odds ratio (OR) per standard deviation (SD) 1.46, 95% CI 1.43, 1.48), Asian (OR per SD 1.68, 95% CI 1.45, 2.06), and Latinx cohorts (OR per SD 1.42, 95% CI 1.29, 1.57), and African ancestry (OR per SD 1.32 95% CI 1.26, 1.38). African ancestry populations had higher average polygenic risk scores compared to other ancestries, and incorporation of *APOL1* risk allele status in this group further increased genetic risk for CKD [1]. Understanding the development of polygenic risk scores is central to understanding their potential clinical utility and limitations in generalising across populations.

Polygenic risk scores derived from GWAS populations, such as UK BioBank, require validation in an independent population before transition to clinical utility. Several polygenic risk scores have been validated for kidney phenotypes including eGFR and CKD status, predominantly from European ancestry, as listed in Table 2. A polygenic risk score for eGFR validated in the ARIC study, with mean age 54.3 years and mean eGFR of 99.6 ml/min/1.73 m^2^, found a hazard ratio (HR) per SD of 1.33 (95% CI 1.28, 1.37) showing that significant variation in eGFR was attributed to genetic risk in a population with normal kidney function [18]. Another validation study in the INTERVAL cohort of 50,000 participants, median age 44, found that per one SD increase in polygenic risk score was associated with − 0.90 (95% CI − 1.45, − 0.36) ml/min/1.73 m^2^ of eGFR [19]. The youngest population studied, an adolescent population in the Netherlands aged 11–22 years, with median eGFR 97.65 ml/min/1.73 m^2^ (IQR 89.28–107.41) found polygenic risk scores explained 5.04% of variability in kidney function [53]. The oldest validation cohort was the ASPREE cohort (aged over 70 years old) which found a clinically meaningful difference between those with high and low polygenic risk scores; those with the highest risk polygenic risk scores had the lowest mean eGFR of 57 ml/min/1.73 m^2^, whereas the lowest risk group had a mean eGFR of 75 ml/min/1.73 m^2^ [16]. The polygenic risk score is static across the lifespan, and validating polygenic risk scores at older age suggests that they are relevant at younger ages, when eGFR is preserved. Polygenic risk scores have also been developed to further understand genetic influences on rate of decline of kidney function. Nine genetic variants were found to associate with a decline in eGFR, with higher heritability estimates of eGFR decline in those with diabetes (1.14%) and CKD (1.48%), which was greater when compared to average risk populations with only 0.51% of decline attributed to polygenic risk scores [17]. Polygenic risk scores for a rapid decline in kidney function phenotype (25% decline in eGFR) carry a 1.29-fold increased risk of CKD, which did not correlate with the risk of kidney failure, but did carry a 1.2-fold increased risk for acute kidney injury [54]. A polygenic risk score for eGFR studied in the German Chronic Kidney disease study found this polygenic risk score was associated with kidney failure (HR 1.22 95% CI 1.12, 1.34), but also myocardial infarction (HR 1.15 95% CI 1.06, 1.25) and mortality (HR 1.12 95% CI 1.04, 1.22), suggesting polygenic risk scores capture multiple genetic pathways that can be involved in other diseases [55, 56]. Taken together, these studies show that polygenic factors explain a proportion of the variability in kidney function across the life span, and are a potential clinical tool to help stratify and screen high-risk patients and target groups at risk of decline.

**Table 2:** Summary of polygenic risk scores validated for kidney disease phenotypes

First author	PMID	Year	PGS	PGS phenotype	Validation population	Number	PGS validation	Comments
Khan	35710995	2022	PGP000269	CKD defined as eGFR < 60 ml/min/1.73m^2^ Control defined as eGFR > 90 ml/min/1.73m^2^	Ancestry European African LatinX Asian All cohorts	97,050 14,544 3625 8625 123,844	OR per SD 1.46 (1.43, 1.48) 1.32 (1.26, 1.38) 1.42 (1.29, 1.57) 1.68 (1.45, 2.06) 1.44 (1.42, 1.57)	Addition of APOL1 allele improved performance in patients of African Ancestry. CKD dichotamised
Yu	34548389	2021	PGS000883	Incident CKD with eGFR of 60 ml/min per 1.73 m^2^ and more than 30% eGFR decline during a follow-up visit compared with baseline	ARIC CKD ESKD Incident AKI	8866	HR per SD 1.33 (1.28, 1.37) 1.24 (1.04, 1.47) 1.05 (1.00, 1.10)	Developed in European ancestry, validated in at-risk populations for kidney disease, and assessed decline in eGFR
Wuttke	31152163	2019	PGS000728	eGFR	UK Biobank Acute renal failure Chronic renal failure	452,264	OR per 10% lower PGS 1.30 (1.16, 1.47) 2.13 (1.90, 2.39)	Most relevant to European cohorts, validates eGFR PGS for acute and chronic kidney disease
Ritchie	3470571	2021	PGS000728	eGFR	INTERVAL	3,307	eGFR (mL/min/1.72 m^2^) per SD increase in PGS − 0.90 (− 1.45, − 0.36)	Shows that higher risk PGS scores are associated with a small reduction in eGFR
Xie	32527150	2020	PGS00303	eGFR	TRAILS	1,354	Variance in eGFR explained 5.04%	Validation in young healthy cohort with preserved kidney function
Gorski	33137338	2021	PGS000664	Decline in eGFR of 3 mL/min/ 1.73 m^2^/year or a decline 25% or more in CKD stage III patients	UK biobank ESKD AKI	6828 16,492	1.01 (087, 1.18) 1.20 (1.08, 1.33)	PGS score used for rapid decline
Gorski	35716955	2022	–	CKD = eGFR < 60 Decline in eGFR ml/min/year	HUNT study AKI ESKD	15,512 6708	OR high vs low risk group 1.27 (1.08, 1.50) 1.35 (1.03, 1.77)	Validates decline in eGFR for outcomes of acute and chronic kidney disease
Steinbrenner	36481179	2022	003377	eGFR	German CKD study Kidney failure	4924	HR for PGS 1.22 (1.12, 1.34)	Validates PGS for eGFR with CKD and other cardiovascular outcomes
Bakshi		2023	PGS000883 PGP000269	As detailed above	ASPREE CKD eGFR CKD stage CKD eGFR CKD stage	11,813	OR per SD 1.71 (1.63, 1.80) 1.36 (1.30, 1.42) 1.48 (1.40, 1.55) 1.23 (1.18, 1.29)	Validates a variety of PGS scores for CKD stages and eGFR

The validated polygenic risk score, relevant kidney phenotypes and key outcomes are described

*ARIC* atherosclerosis risk in community, *TRAILS* tracking adolescents individual lives survey), *ESKD* end-stage kidney disease

Polygenic risk scores are an exciting new tool that provide insight into complex polygenic factors affecting a trait, and correlate well at a population level; however, their usefulness to inform individual risk is limited. A high polygenic risk score does not confer a guaranteed disease state, and a low polygenic risk score predicting low genetic risk is not necessarily a protective factor. Use of polygenic risk scores requires very careful tailoring and validation before integration into clinical practice. Several possible applications include screening high risk groups for progression to CKD, or helping predict those at lower risk of progression to kidney failure. Chronic kidney disease is a heterogeneous disease group, and while a polygenic risk score may detect high-risk genes in common pathways such as fibrosis, it cannot account for more complex genetic influences or environmental factors such as lifestyle factors.

### Polygenic risk scores identifying future research targets

Polygenic risk scores have also been utilised in conjunction with proteomic data to identify potential genes and proteins involved in disease pathogenesis. These studies identified proteins positively associated with eGFR; Testican-2, klotho, carbonic anhydrase-related protein 10, hypoxanthine–guanine phosphoribosyltransferase, and angiostatin. Strongest negative associations with eGFR were found with cystatin c, collagen a-1(XV) and desmocollin-2 [18]. *UMOD* and *TENM3* were identified to be important for patients with diabetes and CKD [57]. *UMOD* encodes Tamm Horsfall protein, and is frequently identified in GWAS for kidney traits. The postulated mechanism is through variant activation of the sodium co-transport, promoting development of hypertension, different to the mechanism in ADTKD-*UMOD* [58]. The function of *TENM3* in the kidney is unknown, but has been associated with CKD in the UK BioBank. Identification of these genes offers possible gene targets for future research in CKD. This suggests that there are shared biological pathways in CKD, despite clinical heterogeneity, which are reflected in the polygenic risk score.

### Polygenic risk scores in targeted disease states

Studies that incorporate more individuals with comorbidities, with higher rates of kidney disease, will strengthen the understanding of the clinical or biological utility of polygenic risk scores, and refine the understanding of the genetic risk for more advanced disease states. However, these studies will also need to consider the way in which these comorbidities can make our interpretation of the genetic factors more difficult. Similar investigation into more targeted causes of kidney disease, such as IgA nephropathy and membranous nephropathy, with established GWAS is warranted as these diseases have significant genetic association [20, 21]. It may also be useful to examine the extent to which genetic risks may compound other common risk factors such as hypertension or diabetes, where family studies have shown possible increased genetic risk.

#### Polygenic factors in IgA nephropathy

IgA nephropathy is the most common glomerulonephritis world-wide, with variable clinical phenotype and course. Family history is positive in 11.6% of patients and is associated with increased risk of end-stage kidney disease, suggesting the genetic component may be associated with worse prognosis [59]. A polygenic risk score for IgA nephropathy was developed in patients with biopsy-proven IgA with 15 SNPs associated with IgA disease. In sensitivity analysis, most of the association was driven by the HLA locus. In UK Biobank patients with haematuria, polygenic risk scores suggested that 19% were potentially related to IgA nephropathy. The discriminatory power of the polygenic risk score between cases and controls was modest, a key limitation of polygenic risk scores [21]. The UK Biobank involves a large population, and while the polygenic risk score is able to predict incident IgA, the clinical relevance may be a milder phenotype as the polygenic risk score was generated for the clinical phenotype of haematuria rather than for biopsy-proven IgA disease. Twenty SNPs associated with IgA in GWAS were studied in Chinese IgA patients and a score to predict progression to kidney failure was generated, which when added to a clinical risk model, improved case discrimination [60]. Polygenic risk scores to assess risk of progression to end-stage kidney failure in IgA have been developed in Asian populations, with polygenic risk scores able to predict clinically relevant phenotypes of kidney failure [60]. Interestingly, models that included HLA genes were more powerful at predicting kidney failure [61]. Importantly, a polygenic risk score that predicts disease progression, or that could be added to the Oxford classification would be an interesting application for IgA polygenic risk scores.

#### Polygenic factors in membranous nephropathy

**Fig. 2:**
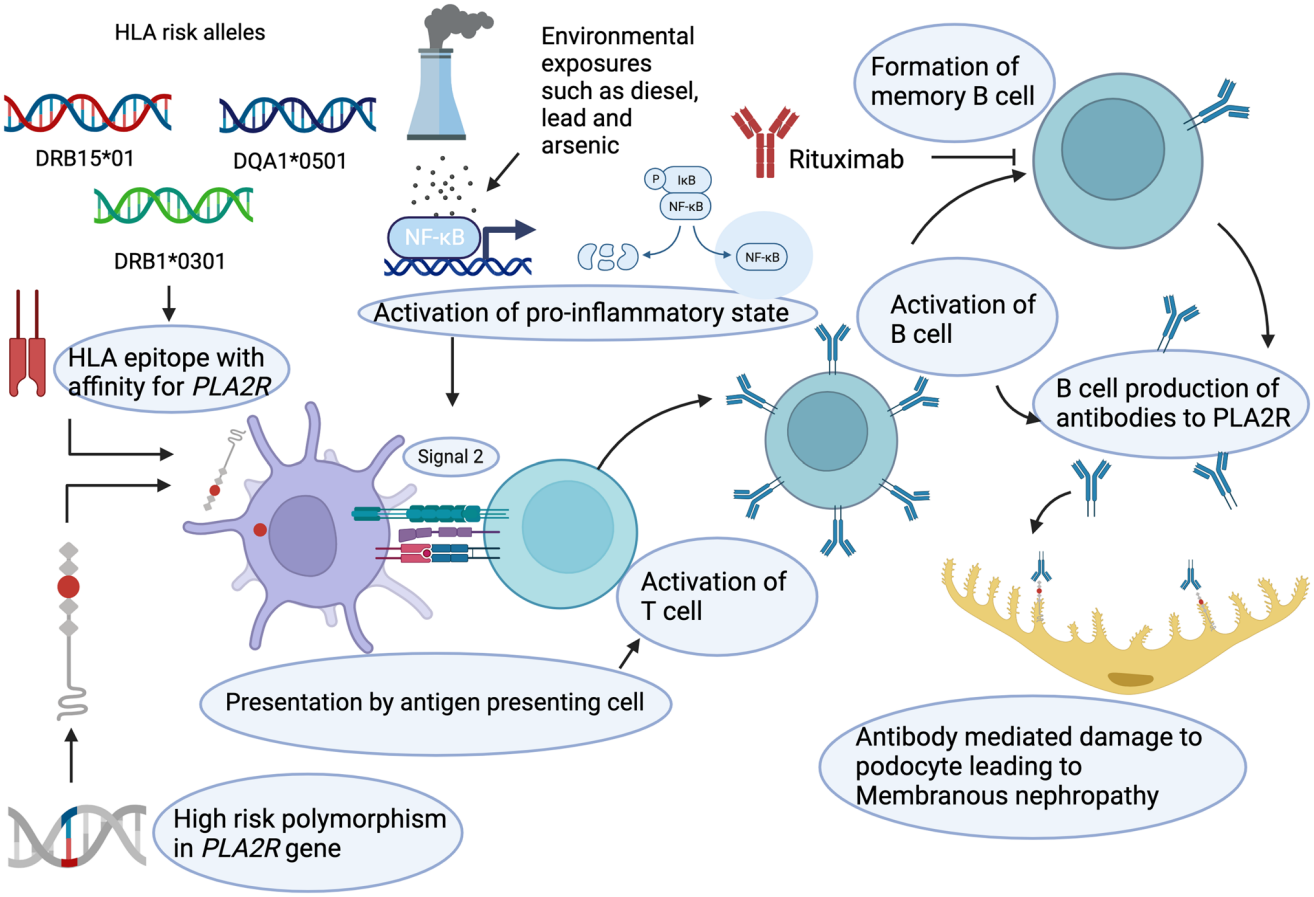
Proposed genetic and environmental factors predisposing to membranous nephropathy. Polymorphisms in HLA alleles lead to generation of major histocompatibility complex (MHC) with a high affinity to certain PLA2R epitopes. Polymorphisms in the PLA2R gene produce variations in the protein structure which are presented by the antigen-presenting cell, and interact strongly with the MHC molecules. Environmental triggers, such as lead, arsenic and diesel, trigger activation of the inflammatory pathway, with genetic polymorphisms in NFKB leading to increased inflammatory response, which acts as signal two in the immune activation. T cell activation leads to B cell activation and generation of antibodies to PLA2R, which bind the immunogenic PLA2R on the podocyte, leading to development of nephrotic syndrome in membranous nephropathy. Created with BioRender.com

Membranous nephropathy is the most common cause of adult nephrotic syndrome, with both genetic and environmental associations. Genome-wide association studies have found 25–32% of membranous nephropathy is genetically determined, with key genes being *PLA2R*, encoding the pathogenic podocyte autoantigen, HLA genes, and immune pathway genes (*NFKB1* and *IRF4*). Interestingly, the HLA genes vary by ancestry with risk alleles in DRB1*1501 being predominant in East Asians, DQA1*0501 in Europeans and DRB1*0301 in both ancestries [20]. This study also found an interaction between HLA risk haplotypes and the *PLA2R* SNP; in East Asians there was a heightened risk with an OR 88.8 (95% CI 28.0, 270.3) and in Europeans there was an OR 14.1 (95% CI 10.0, 22.1). Results from the GWAS were used to create a polygenic risk score for membranous nephropathy, with genetic factors attributed to predict 29% of disease. This was validated in well characterised populations, finding the membranous nephropathy polygenic risk score able to discriminate from other common glomerular diseases including IgA and focal segmental glomerulosclerosis. Diagnosis of membranous nephropathy based on serological testing of PLA2R by ELISA has high specificity (99–100%) but low sensitivity (51–60%), and in cases where ELISA was negative (< 2 U/mL) or unclear (2–20 U/mL), incorporation of the polygenic risk score aided diagnostic clarity in 20–37% of cases, with 99% specificity, supporting clinical utility of targeted polygenic risk score disease states. Membranous nephropathy is also associated with environmental exposures such as lead and arsenic, in a French population [62]. In Chinese populations, epidemiological data suggest that increasing environmental air exposure is related to increased incidence in both membranous and IgA nephropathy [63]. Animal studies have shown that diesel particulates increase *NFKB* expression and are associated with inflammation in the kidney [64]. Together, this information suggests that genetically predisposed individuals with susceptible polygenic risk state exposed to key environmental factors are primed to activate the immune system and develop membranous nephropathy, as shown in Fig. 2 [65]. Polygenic risk scores may improve diagnostic utility and offer areas for further research and treatment targets.

#### Hypertension

Hypertension affects millions of people world-wide and is an important risk factor for CKD, with strong environmental and genetic factors, and having both monogenic and polygenic causes. Monogenic causes of hypertension are rare but clinically significant, such as Liddle’s syndrome, with an autosomal dominant gain-of-function in the amiloride sensitive chloride channel, with a prevalence of 1.52% in young patients with unexplained early onset hypertension [66]. Family studies show that hypertension clusters in families, and population studies estimate heritability at 17–52% depending on the population studied [10, 67]. Genome-wide association studies identified 900 loci associated with hypertension, although they explain only 2.2% of the variance in blood pressure, with each individual locus explaining an extremely small degree of difference in blood pressure [22]. The large polygenic risk score for hypertension, including 28 key loci, had an OR of 1.65 per SD for systolic blood pressure, which also demonstrated an association with coronary artery disease and stroke, but interestingly not with chronic kidney disease or albuminuria [22]. A polygenic risk score for hypertension was generated from large multi-ethnic cohorts which showed higher average polygenic risk scores in participants of African ancestry compared to European ancestry, and found high polygenic risk scores (90–100th centile) were associated with hypertension with an OR of 2.07 for African ancestry and an OR of 1.43 for European ancestry. This polygenic risk score was also associated with hypertension (OR 1.45 95% CI 1.41, 1.45), coronary artery disease (OR 1.13 95% CI 1.07, 1.18), type 2 diabetes (OR 1.19 95% CI 1.13, 1.24), chronic kidney disease (OR 1.13 95% CI 1.01, 1.26), obesity (OR 1.09 95% CI 1.06, 1.12) and ischaemic stroke (OR 1.15 95% CI 1.04, 1.28) [68]. Despite the strong polygenic risk associated with hypertension, lifestyle factors are likely to be more important. A study of 277,005 individuals from the UK Biobank assessed lifestyle factors including diet, smoking, alcohol consumption, sedentary behaviour, BMI and urinary sodium excretion, and found that those with high genetic risk and unhealthy lifestyle had higher SBP of 146 mmHg and 142 mmHg with an unfavourable lifestyle, whereas those with low genetic risk and unfavourable lifestyle had SBP of 140 mmHg, suggesting that low polygenic risk cannot mitigate the influence of lifestyle factors [69]. A possible use of polygenic risk scores could be to predict response to anti-hypertensive treatment in order to guide therapy. A GWAS generated from the ‘Genetics of Drug Responsive in Essential Hypertension’ and ‘Losartan Intervention for Endpoint Reduction in Hypertension’ study subjects, enriched with hypertension cases, studied response to four categories of anti-hypertensives; diuretics, beta-blockers, calcium channel blockers and angiotensin blockade stratified by low and high risk polygenic risk scores. The study was unable to identify individual drug responses, but did find that individuals who had difficulties controlling hypertension had higher polygenic risk scores for hypertension [70]. Clinical applications of polygenic risk scores represent an area of ongoing research.

#### Novel potential applications of GWAS and polygenic risk scores in clinical practice

Polygenic factors are being applied in many novel aspects of medicine, including in disease management approaches. A recent study in patients aged 31 (26–28) years undergoing chemotherapy found that utilising a polygenic risk score for eGFR was an independent predictor of cisplatin levels, suggesting it may be another modality to consider kidney function across life span [71]. Solute clearance in peritoneal dialysis is associated with five key SNPs from GWAS and may offer insight into assessment of peritoneal dialysis suitability [72]. Pharmacogenomics to identify gene-drug interactions is another potential application, and early feasibility studies show promising clinical utility in CKD management, such as genetic variation in *CYP2C9* association with impaired metabolism of losartan and uncontrolled hypertension. The important clinical application was that 36% of community general practitioners utilised pharmacogenomic data to guide hypertensive treatment [73]. Living related kidney donation is a unique aspect of kidney health and is a potential area for pre-emptive genetic testing. Cascade testing of potential living related donors for a family member with kidney failure has a clear benefit in assessing risk of kidney disease in live donors [74]. Assessing polygenic risk in potential living donors is less clear, however recommendations suggest considering screening potential living donors of African descent for *APOL1* risk alleles. Current consensus opinion recommends potential living kidney donors who report African ancestry to be informed about *APOL1* and risk of kidney failure, and appropriate counselling and testing should be offered to those with risk factors for kidney failure [43]. Results of the *APOL1* long-term Kidney Transplantation Outcomes Network (APOLLO) will better inform potential donors on possible longitudinal risk [75]. Currently, the role of polygenic risk scores in this area is yet to be defined, but still a potential application, albeit unrefined at this time [76]. Incorporating genetic data into clinical practice is an exciting and developing field.

## Conclusions

Kidney function is determined by monogenic and polygenic factors with important interacting environmental determinants. Polygenic risk scores are able to stratify high and low risk groups for kidney disease at a population level but are not deterministic for individual kidney function, where environmental and lifestyle factors are potentially more relevant. Polygenic risk scores at a population level are useful to assess risk, but their role in guiding individual practice is less clear and not ready for integration into individual patient care. Potential applications include guiding prognosis in targeted disease states such as IgA nephropathy. Of course, an exciting application is the identification of new therapeutic targets in common pathways.
